# Multilocus re-evaluation of species boundaries in the Central European *Psilopteryx psorosa* species group (Trichoptera, Limnephilidae) with shallow morphological differentiation

**DOI:** 10.3897/zookeys.1283.193317

**Published:** 2026-07-01

**Authors:** Michaela Šamulková, Tomáš Navara, Pavel Chvojka, Zuzana Čiamporová-Zaťovičová, Patrik Macko

**Affiliations:** 1 ZooLab, Department of Biodiversity and Ecology, Plant Science and Biodiversity Centre, Slovak Academy of Sciences, Dúbravská cesta 9, 845 23 Bratislava 4, Slovakia ZooLab, Department of Biodiversity and Ecology, Plant Science and Biodiversity Centre, Slovak Academy of Sciences Bratislava Slovakia; 2 Institute of Zoology, Slovak Academy of Sciences, Dúbravská cesta 9, 845 06 Bratislava 4, Slovakia Department of Entomology, National Museum Prague Czech Republic; 3 Department of Entomology, National Museum, Cirkusová 1740, 193 00 Prague 9, Czech Republic Department of Ecology, Faculty of Natural Sciences, Comenius University in Bratislava Bratislava Slovakia; 4 Department of Ecology, Faculty of Natural Sciences, Comenius University in Bratislava, Ilkovičova 3278/6, 841 04 Bratislava 4, Slovakia Institute of Zoology, Slovak Academy of Sciences Bratislava Slovakia

**Keywords:** Integrative taxonomy, MinION Nanopore, mtDNA, population analyses, species delimitation, *wingless*

## Abstract

The caddisfly genus *Psilopteryx* Stein, 1874 (Limnephilidae) represents a taxonomically challenging group in which species boundaries have traditionally been defined solely by adult morphology, as larval stages lack reliable diagnostic characters. This difficulty is particularly evident within the *Psilopteryx psorosa* species group in Central Europe, where species delimitation has relied primarily on subtle and often variable differences in male genital morphology, while reliable diagnostic characters for larvae and females remain unavailable. In this study, we applied a multilocus molecular approach to evaluate species boundaries within this complex. We analysed 58 specimens collected across eight montane regions in the Czech Republic, Slovakia, and Germany, including material from the type locality of *P. psorosa*. Based on male genital morphology and following the taxonomic concept of Mey and Botosaneanu (1985), the analysed specimens were initially assigned to two subspecies: *P. psorosa psorosa* and *P. psorosa bohemosaxonica*. Two mitochondrial genes (COI, cytB) and one nuclear marker (*wingless*) were analysed using haplotype-based analysis, genetic distances, and phylogenetic inference. Across all analysed markers, genetic divergence was low, with median uncorrected *p*-distances not exceeding 0.3% and maximum divergence below 2%. Mitochondrial haplotypes were widely shared among regions and taxa, and neutrality tests performed on the complete mtDNA dataset yielded significantly negative values. Haploweb analysis of the nuclear marker recovered a single field for recombination (FFR), while both single-gene phylogenetic reconstructions and the concatenated multilocus analysis failed to recover reciprocally monophyletic groups corresponding to the traditionally recognised taxa. Our results indicate that the analysed forms represent a single evolutionary lineage and that the observed morphological differentiation reflects geographically structured intraspecific variation rather than distinct species.

## Introduction

With more than 17,000 described species across 65 extant families, caddisflies (Trichoptera) are among the most diverse lineages of aquatic insects worldwide (Morse 2026). They are distributed across all major zoogeographic regions sensu Wallace (1876), with approximately half of all known species occurring in the Oriental (>5,800 species; Morse et al. 2019) and the Neotropical region (>3,300 species; De Moor and Ivanov 2008). In the Palearctic, and Europe in particular, the caddisfly fauna is considered relatively well studied, with ~ 1,800 species currently recognised (see Kučinić et al. 2025 and references therein). Nevertheless, taxonomic research remains intense, as evidenced by the description of more than 360 new European species during the first two decades of the 21^st^ century (Sánchez-Campaña et al. 2023).

Despite the description of many species and the long history of taxonomic research, caddisfly systematics remains burdened by persistent difficulties in species delimitation (for a recent synopsis, see Frandsen et al. 2025). Species-level taxonomy relies predominantly on male genitalic characters that are highly diverse and shaped by sexual selection (Eberhard 2015), while their interpretation has accumulated a long history of inconsistent terminology and unresolved homology across the literature (e.g., Nielsen 1957; Ross 1963; Tuxen 1970; Marshall 1979; Oláh and Johanson 2008). In contrast, females are often associated with males only indirectly, and larval identification is typically restricted to final instars and based on subtle or variable characters (Schefter and Wiggins 1987; Wiggins 2004). Such limitations may be particularly pronounced in cold-adapted montane taxa, where long-term environmental stability and historical range fragmentation have promoted strong geographic population structuring (Hewitt 2000; Schmitt 2007; Finn et al. 2011). In such systems, morphological divergence remains relatively small compared to genetic divergence, potentially complicating species delimitation based solely on phenotypic traits (Bickford et al. 2007; Padial et al. 2010; Vuataz et al. 2016).

The genus *Psilopteryx* Stein, 1874 (Limnephilidae) exemplifies this problem, particularly in the *Psilopteryx psorosa* species group of the Carpathians (Oláh et al. 2015). In this group, populations inhabiting cold montane streams exhibit geographically structured subspecific variation shaped by Pleistocene glacial isolation, while morphological differentiation remains comparatively limited (Mey and Botosaneanu 1985). *Psilopteryx psorosa* (Kolenati, 1860) was originally described in the genus *Chaetopteryx* Stephens, 1829 as *Ch. psorosa* Kolenati, 1860 from the Jeseníky Mountains (Sudetes) (Kolenati 1860) and later became the type species of the genus *Psilopteryx* Stein, 1874 by monotypy (Stein 1874). At the turn of the nineteenth and twentieth centuries, the species was recorded from the Sudetes and the Northern and Eastern Carpathians in the territory of present-day Czech Republic, Slovakia, Poland, and Ukraine (Dziędzielwicz 1883, 1884, 1891, 1908, 1920; Pongrácz 1919; Pax and Maschke 1935; Mayer 1936). Long-term geographic isolation led to differentiation in male genital morphology, which resulted in the description of *P. carpathica* Schmid, 1952, and subsequently also in the recognition of geographically structured subspecies within *P. psorosa* sensu lato (Botoșăneanu and Schneider 1978).

Mey and Botosaneanu (1985) re-evaluated this variation across the entire distribution range and recognised six allopatrically distributed subspecies, documenting gradual geographic change and partial overlap of diagnostic characters. Szczęsny (1987) subsequently pointed out the inadequate redefinition of *P. p. psorosa* and documented variation within North Carpathian populations. More recently, Oláh et al. (2015) elevated several of these subspecies to species rank and described additional taxa, defining a *P. psorosa* species group based primarily on fine-scale differences in male genital morphology. The subtle, partly continuous nature of these characters, together with recurrent misidentifications reported from Central Europe, raises the question of whether the recognised taxa represent independently evolving lineages or geographically structured population-level variation. Neu et al. (2018) consider the distribution of species and subspecies of the *P. psorosa* group to be unclear and suggest further investigations. Altogether, 17 species of *Psilopteryx* are currently listed from Central Europe, the Carpathians, the Balkans, and Anatolia (Morse 2026), highlighting the taxonomic and biogeographic complexity of the genus across montane regions.

Given the limited and partly overlapping morphological differentiation, molecular data provide an independent framework for evaluating species boundaries (Dayrat 2005; Padial et al. 2010; Fujita et al. 2012). Within an integrative taxonomic framework, multilocus approaches combining mitochondrial and nuclear markers have proven particularly effective in disentangling shallow population structure from species-level divergence in morphologically conservative or recently diversified insect groups (e.g., Talavera et al. 2022; Zhou et al. 2022; Kilian et al. 2022). In Trichoptera, multilocus phylogenetic and barcoding studies have revealed cryptic diversity and clarified species limits in lineages such as the subfamily Drusinae (Vitecek et al. 2015, 2020), underscoring the importance of molecular evidence in groups characterised by limited structural divergence. Nevertheless, molecular data are most informative when interpreted in conjunction with morphology, geography, and evolutionary history (e.g., Pauls et al. 2006; Hrivniak et al. 2020). Despite the long taxonomic history of *Psilopteryx*, no comprehensive molecular assessment has yet been conducted for the *P. psorosa* species group, leaving its evolutionary structure and species boundaries largely untested.

This study focuses on the Central European portion of the *P. psorosa* species group, a region characterised by the occurrence of four taxa that Oláh et al. (2015) consider to be species, i.e. *P. psorosa* (Kolenati), *P. bohemosaxonica* Mey & Botosaneanu, and the recently described taxa *P. harmas* Oláh & Chvojka, 2015 and *P. javorensis* Oláh, 2015 (Oláh et al. 2015). However, the analysed material originated exclusively from the known distribution ranges of only two taxa with broader distribution, namely *P. psorosa psorosa* (Kolenati) and *P. psorosa bohemosaxonica* Mey & Botosaneanu, (1985) in Bohemia, adjacent territory of Bavaria, northern Moravia, and western and northern Slovakia, whereas specimens from the distribution areas and type localities of *P. harmas* (Eastern Carpathians) and *P. javorensis* (Poľana and Javorie Mountains, central Slovakia) were not included in the present study. We analysed multilocus sequence data from 58 *Psilopteryx* individuals collected predominantly in the Czech Republic and Slovakia, including material from the type locality of *P. psorosa* (Hrubý Jeseník Mountains, Sudetes) and from a site situated near the type locality of *P. bohemosaxonica* in the Ore Mountains. Using two mitochondrial genes and one nuclear marker, we evaluated phylogenetic relationships, haplotype structure, and levels of genetic divergence. We tested whether the taxa currently recognised within the analysed region represent multiple independently evolving lineages or a single, morphologically variable species, aiming to provide an integrative molecular framework for the ongoing taxonomic revision of this complex.

## Materials and methods

### Specimen sampling and morphological identification

The analysed material comprised 58 adult specimens of the genus *Psilopteryx*, collected in 2010 and between 2021 and 2023 at 23 sampling sites across eight montane regions in Slovakia, the Czech Republic, and Germany (Suppl. material 1). Specimens of *Psilopteryx* were collected by sweeping riparian vegetation using a standard sweep net during surveys of caddisfly assemblages. Specimens were identified using the available identification atlas (Malicky 2004). For consistency, individuals were assigned following the traditional subspecific concept of Mey and Botosaneanu (1985), rather than the subsequent species-level interpretation of Oláh et al. (2015), prior to molecular analyses. Specimens were preserved in 96% ethanol and stored at −25 °C until further processing.

### DNA extraction

For molecular analyses, DNA was extracted from each specimen using the Chelex protocol (Casquet et al. 2012). For each sample, 150 µl of Nuclease-Free Water (NFW), 10 µl of Proteinase K (Canvax), and 0.015 g of Chelex 100 (Sigma-Aldrich) were used. DNA was extracted from the second and third legs from one side of the body and a small piece of thoracic tissue. The tissue was first dried to remove the fixative, then immersed in the Chelex solution. Samples were incubated for 6 h at 55 °C.

### PCR amplification

Two mitochondrial (mtDNA) genes were amplified: the standard DNA barcode marker cytochrome c oxidase subunit I (COI – primers LCO1490-JJ and HCO2198-JJ; ~657 bp; Astrin and Stüben 2008) and cytochrome b (cytB – primers CB3 and CB4; ~351 bp; Barraclough et al. 1999). The nuclear gene *wingless* (wg – primers Wg550f and WgAbRz; ~450 bp; Wild and Maddison 2008) was also amplified. Detailed PCR protocols and primer sequences for each fragment are provided in the Suppl. material 2. Amplification success was verified by electrophoresis on 1% agarose gels.

### Library preparation, sequencing, and bioinformatic processing

The three amplicons (COI, cytB, and wg) from each specimen were pooled in equimolar concentrations. Each sample was assigned a unique barcode sequence to enable demultiplexing, and a sequencing library was prepared following the Ligation Sequencing of Amplicons – Native Barcoding Kit 96 V14 protocol (SQK-NBD114.96; Oxford Nanopore Technologies, Cambridge, UK). After adapter ligation, the purified library was quantified fluorometrically (Quantus™ Fluorometer, Promega) and diluted to the recommended concentration for loading onto the flow cell. Sequencing was performed on a MinION Mk1B platform using FLO-MIN114 (R10.4.1) flow cell (ONT, Cambridge, UK). Run management was conducted using MinKNOW v. 22.05.05, which simultaneously performed basecalling and demultiplexing via the integrated barcoding module, generating separate directories of raw FASTQ files for each specimen.

Subsequent processing was performed on each sample individually in Linux. The quality of raw reads was assessed using NanoPlot (v. 1.46.1; De Coster and Rademakers 2023). Residual adapter sequences were removed using Porechop (v. 0.2.4; Wick et al. 2017). Reads were then filtered with NanoFilt (v. 2.8.0; De Coster et al. 2018) using a minimum quality score of Q ≥ 12 and a minimum length of 150 bp. Consensus sequences were generated using Amplicon Sorter (Vierstraete and Braeckman 2022), which sorts amplicons in a reference-free manner based on sequence length and similarity. Clusters were separated at a threshold of ~ 95–96%, representing the limit for distinguishing closely related lineages within a sample. The output consisted of consensus sequences for individual specimens, which were subsequently assigned to their corresponding amplicon (COI, cytB, or wg). To this end, all consensus sequences for each sample were individually analysed using BLASTn (NCBI) and each sequence was assigned to the appropriate gene fragment based on the highest BLAST similarity. In cases where multiple alternative consensus sequences were obtained for a single fragment, the consensus with the highest read count was selected as the representative sequence for downstream analyses. For the nuclear marker wg, heterozygous positions retained in the consensus sequences as ambiguous nucleotides were subsequently phased using the PHASE algorithm implemented in DnaSP (Rozas et al. 2017).

Validated consensus sequences were organised into three FASTA files, each containing sequences from all analysed specimens for a gene (COI, cytB, or wg). For each marker, sequences were aligned using the MUSCLE algorithm (Edgar 2004) in MEGA (v. 12.1; Kumar et al. 2018). Aligned nucleotide sequences were subsequently translated into amino acids to verify reading-frame integrity and to exclude the presence of stop codons or other indications of pseudogenes or consensus errors. The resulting sequence dataset was uploaded to the Barcode of Life Data System (BOLD) with complete metadata, including specimen information (e.g., collection locality, geographic coordinates, collection date, and taxonomic identification). The dataset is available under doi: https://doi.org/10.5883/DS-TRIPSILO (dataset will be made publicly available upon acceptance). The sequences were also deposited in GenBank (accession numbers will be provided upon acceptance).

### Population genetic analyses and phylogenetic inference

Genetic variability was initially assessed in MEGA using uncorrected *p*-distances with pairwise deletion and 500 bootstrap replicates and visualised as boxplots to compare variability among markers. For the mtDNA genes COI and cytB, aligned sequences were collapsed into unique haplotypes in FaBox 1.61 (Villesen 2007), and median-joining networks (Bandelt et al. 1999) were constructed in POPART 1.7 (Leigh and Bryant 2015). Neutrality tests for these mtDNA markers were performed on the complete dataset including all analysed individuals in Arlequin v. 3.5 (Excoffier and Lischer 2010) using Tajima’s D and Fu’s Fs statistics, with significance assessed by 10,000 coalescent simulations. For the nuclear marker wg, heterozygous positions were phased in DnaSP v. 5 (Rozas et al. 2017) using the PHASE algorithm. The inferred haplotypes were subsequently analysed in HaplowebMaker (Spöri and Flot 2020). Fields for recombination (FFRs) were identified from allele co-occurrence in heterozygous individuals and used to evaluate conspecificity among morphologically defined taxa.

Each gene fragment (COI, cytB, and wg) was first analysed phylogenetically as an independent dataset using two complementary approaches: Maximum Likelihood (ML) and Bayesian inference (BI). ML analyses were conducted in MEGA, incorporating outgroup sequences retrieved from GenBank. For the COI dataset, *Chaetopteryx atlantica* Malicky, 1975 (GenBank accession number TXW9PJ9M016) and *Pseudopsilopteryx zimmeri* (McLachlan, 1876) (TXWCGT6D014) were used as outgroups. For cytB, *Limnephilus decipiens* (Kolenati, 1848) (TXW37HYF014) and *Pseudopsilopteryx zimmeri* (TXW80UBP014) were included. For the nuclear marker wg, *Chaetopteryx fusca* Brauer, 1857 (TXWEEU94014) and *Chaetopteryx major* McLachlan, 1876 (TXWG882A014) served as outgroup taxa. The best-fitting nucleotide substitution model for each dataset was selected using both the Bayesian Information Criterion (BIC) and Akaike Information Criterion (AIC), which yielded congruent model selections for all datasets. The HKY + I model was selected for COI and cytB, whereas the JC model was selected for wg. Node support was assessed using 500 bootstrap replicates. Bayesian phylogenetic analyses were performed in BEAST v. 2.7.5 (Bouckaert et al. 2014). The substitution models selected in MEGA were applied to the corresponding datasets. A strict molecular clock was implemented for all analyses, and the tree prior was set to the Coalescent Constant Population model, reflecting the low sequence divergence observed among samples. For all single-gene datasets, four independent Markov Chain Monte Carlo (MCMC) runs were performed for 10,000,000 generations, with trees sampled every 1,000 generations. Convergence and effective sample size (ESS) values were assessed in Tracer v. 1.7.1 (Rambaut et al. 2018). The two best independent runs with ESS values above 200 were combined using LogCombiner v. 2.7.5., applying a 10% burn-in. Maximum clade credibility (MCC) trees were generated in TreeAnnotator v. 2.7.5. Final trees were visualised and edited in FigTree v. 1.4.3 (http://tree.bio.ed.ac.uk/software/figtree) and graphically refined in Paint 3D. In addition to single-gene analyses, a combined multilocus dataset was analysed in BEAST under the same substitution models and tree prior settings to obtain a consensus phylogenetic hypothesis across markers. The combined multilocus dataset was analysed for 20,000,000 generations, with sampling every 2,000 generations. Because complete sequences for all three loci were not available from a single outgroup individual, two chimeric outgroup sequences were constructed by concatenating corresponding gene fragments derived from different specimens of the above-mentioned outgroup species. This approach was used solely to provide phylogenetic rooting and does not affect ingroup topology.

## Results

**Figure 1.**
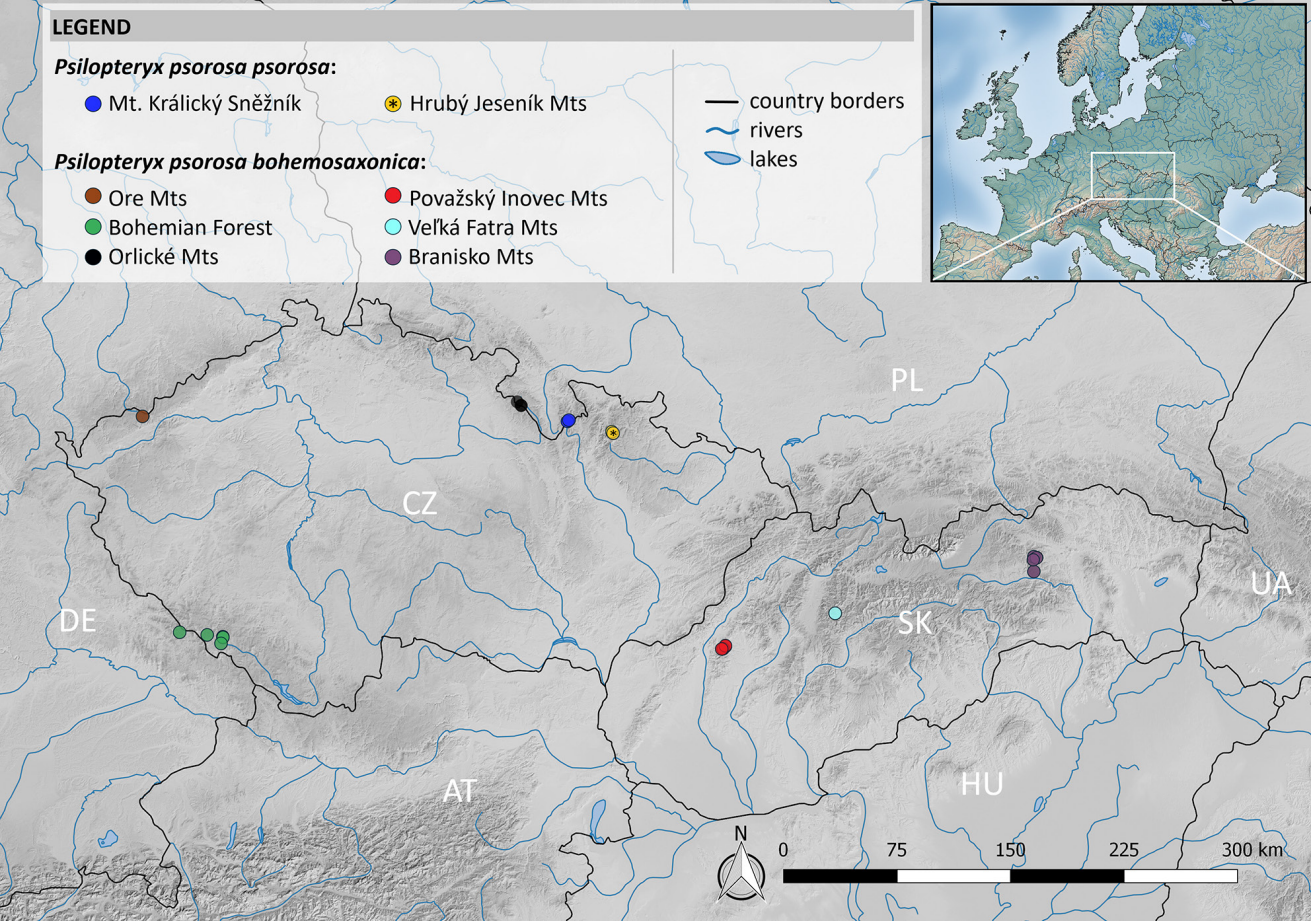
Geographic distribution of the two analysed subspecies of the *Psilopteryx psorosa* species group across Central European mountain ranges. An asterisk (*) denotes the type locality. Country abbreviations: CZ = Czech Republic, SK = Slovakia, PL = Poland, DE = Germany, AT = Austria, HU = Hungary, UA = Ukraine (maps were created in QGIS 2.18).

Based on morphological identification following Mey and Botosaneanu (1985), the analysed material was assigned to two traditionally recognised subspecies: *Psilopteryx psorosa psorosa* and *P. psorosa bohemosaxonica*. Specimens attributed to *P. p. psorosa* originated from the Mt. Králický Snežník and Hrubý Jeseník Mts, including individuals from the type locality region in the Hrubý Jeseník Mts. Specimens assigned to *P. p. bohemosaxonica* were collected from the remaining sampled mountain regions in Central Europe (Fig. 1).

**Figure 2.**
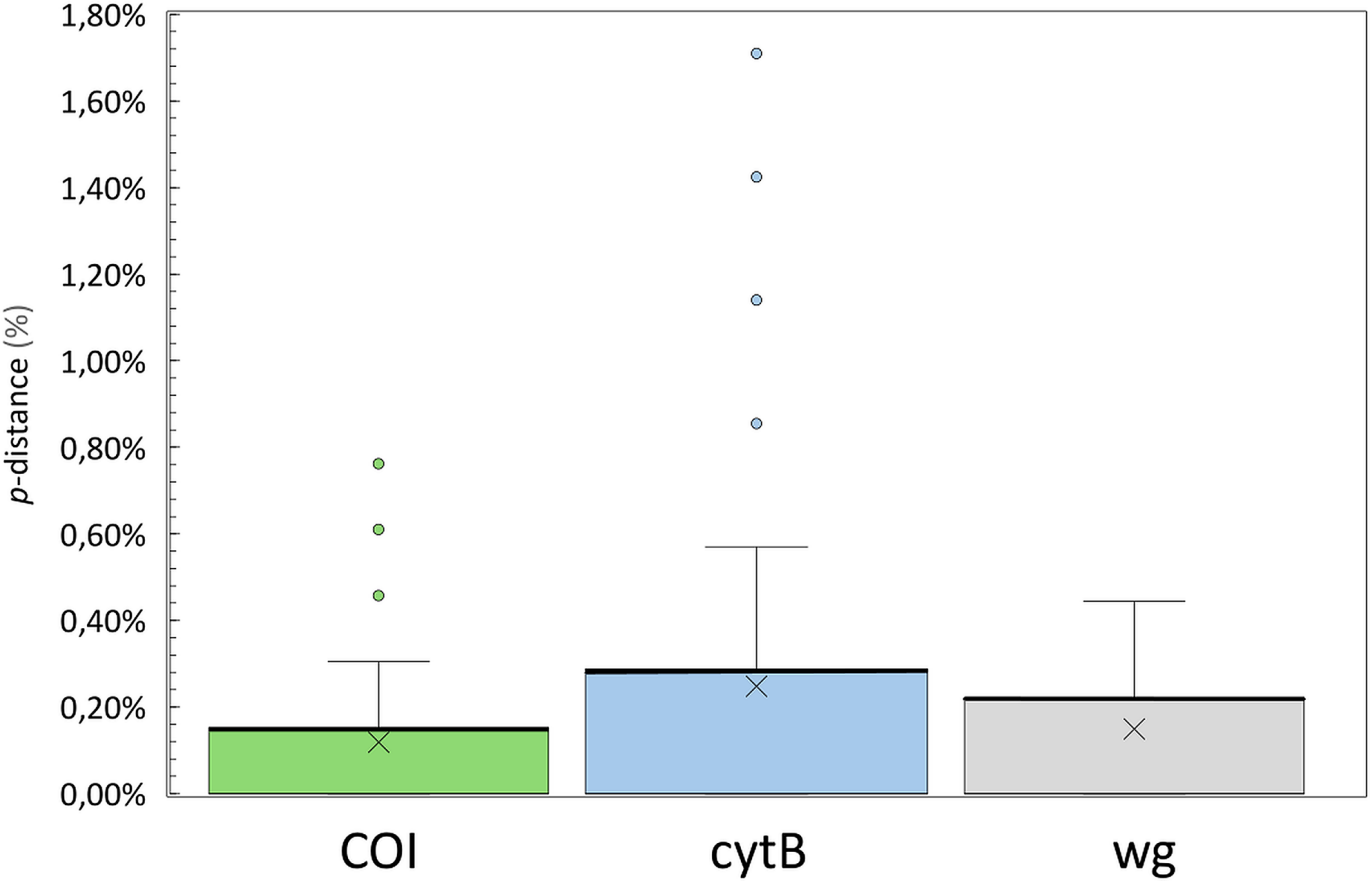
Genetic variability of the analysed markers (COI, cytB, and wg) based on uncorrected *p*-distances. Boxes represent the interquartile range (IQR), bounded by the first (Q1) and third (Q3) quartiles. The horizontal black line indicates the median, and the X denotes the mean. Whiskers extend to values beyond Q1 and Q3, and circles indicate outliers.

Across all analysed markers, genetic variability was low (Fig. 2). Median uncorrected *p*-distances were 0.152% for COI, 0.285% for cytB, and 0.222% for wg (including ambiguous sites), while mean values were 0.119%, 0.248%, and 0.149%, respectively. Maximum observed divergence reached 0.76% in COI, 1.71% in cytB, and 0.44% in wg. Pairwise distances overlapped between individuals assigned to both morphologically defined subspecies, with no clear genetic discontinuity detected.

**Figure 3.**
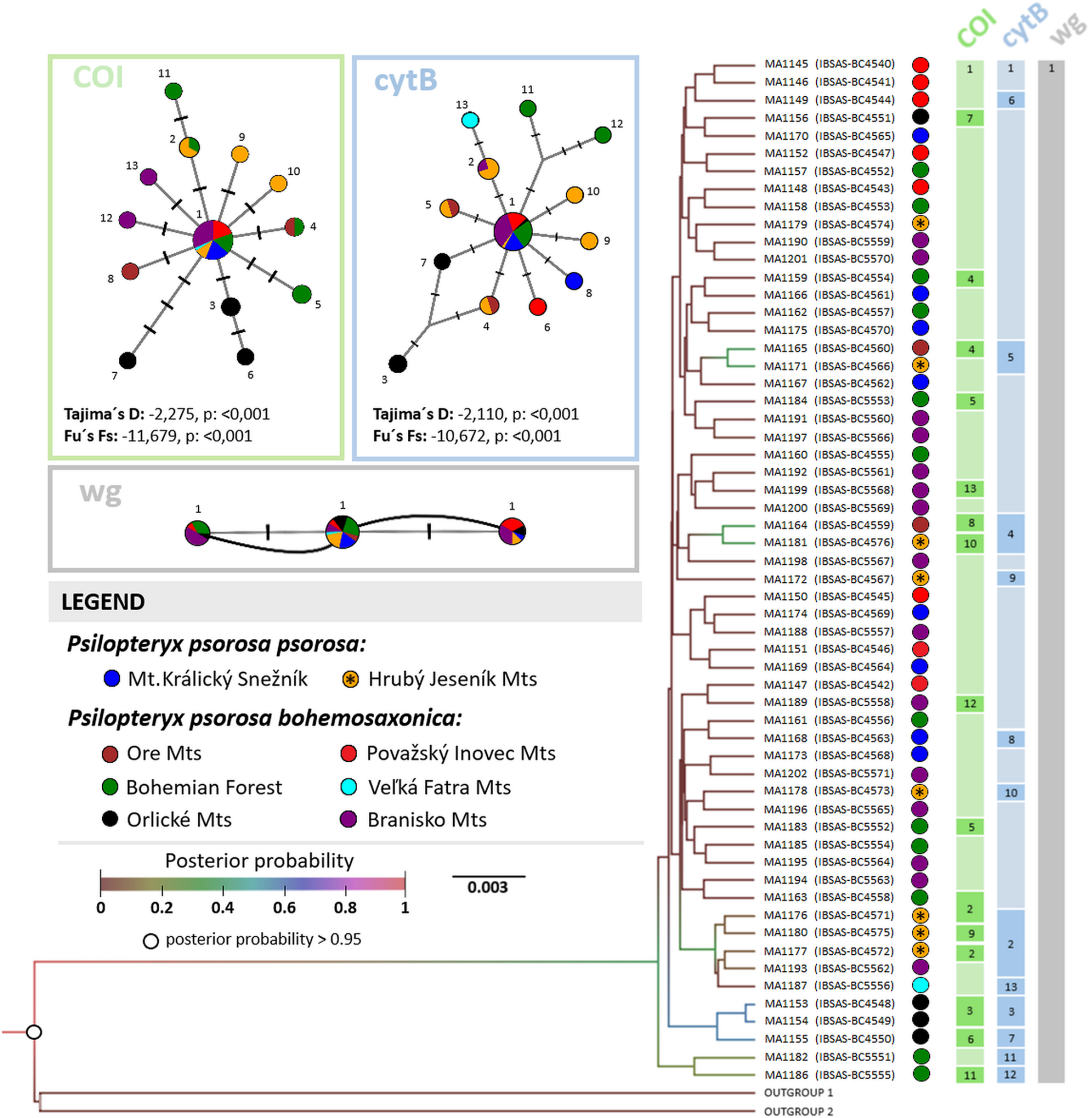
Multilocus Bayesian phylogeny of the morphologically defined subspecies *Psilopteryx psorosa psorosa* and *P. p. bohemosaxonica* based on concatenated COI, cytB, and wg sequences. Adjacent bars indicate haplotype numbers and asterisks mark specimens from the type locality. Codes prefixed with “MA” correspond to specimens analysed in this study, whereas codes in parentheses indicate BOLD Sample IDs. Haplotype networks for COI and cytB and a Haploweb for wg are shown alongside the phylogeny. Connections between alleles in heterozygous individuals define a field for recombination (FFR).

For the mtDNA marker COI, 13 haplotypes were identified among 58 individuals (Fig. 3). The haplotype network displayed a predominantly star-like structure centred on the most frequent haplotype (HT1). Specimens from the Hrubý Jeseník Mts (type locality) were not restricted to a single unique haplotype: individuals from this region occurred in the central and most frequent haplotype (HT1) and shared haplotype HT2 with specimens from the Bohemian Forest. Only HT9 and HT10 represented distinct haplotypes restricted to single individuals. The most divergent haplotype was HT7, differing by three mutational steps from the central haplotype HT1. Overall, haplotypes were shared among individuals assigned to both subspecies and across multiple mountain regions. Similarly, cytB yielded 13 haplotypes. The network topology was shallow and reticulate, with no clear geographic or taxonomic structuring. Individuals from the Hrubý Jeseník Mts co-occurred with specimens from other regions in four haplotypes (HT1, HT2, HT4, and HT5). As in COI, haplotypes were shared between individuals assigned to both morphologically defined subspecies. The distribution of haplotypes differed between the COI and cytB markers, and no consistent mtDNA haplotype structure was observed across the two datasets. For the nuclear marker wg, three haplotypes were identified. Haploweb analysis recovered a single FFR, as all haplotypes were interconnected through heterozygous individuals. No subdivision corresponding to the two morphologically defined subspecies was detected. Neutrality tests performed across all analysed individualsrevealed significantly negative Tajima’s D and Fu’s Fs values (COI: D = −2.275, Fs = −11.679; cytB: D = −2.110, Fs = −10.672; p < 0.001 for both markers). These strongly negative values are consistent with demographic expansion or purifying selection.

Phylogenetic reconstructions using both Maximum Likelihood and Bayesian inference resulted in shallow topologies characterised by short internal branches and generally low statistical support (Suppl. materials 3, 4). Individuals assigned to *P. p. psorosa* and *P. p. bohemosaxonica* were intermingled in all single-gene trees, and no consistent clustering according to morphological assignment or geographic origin was recovered. The multilocus BEAST analysis similarly revealed a poorly structured topology with low posterior probabilities across most internal nodes (Fig. 3). A single subclade with limited posterior support was recovered (pp > 0.5), grouping individuals corresponding to COI haplotypes HT3 and HT6 and cytB haplotypes HT3 and HT7. This cluster corresponded to specimens morphologically assigned to *P. p. bohemosaxonica* from the Orlické Mts. Apart from this limited grouping, internal relationships remained weakly resolved. No reciprocally monophyletic groups corresponding to *P. p. psorosa* and *P. p. bohemosaxonica* were observed, and specimens from the type locality region did not form a distinct lineage.

## Discussion

Our multilocus analyses did not recover genetic structuring corresponding to the two morphologically defined subspecies (*P. p. psorosa* and *P. p. bohemosaxonica*), within the *P. psorosa* group. These taxa were originally distinguished based on subtle differences in male genital morphology (Mey and Botosaneanu 1985) and later elevated to species rank by Oláh et al. (2015). Genetic divergence across all analysed markers was very low, and haplotypes were widely shared among sampled geographic regions. Neither mtDNA markers nor nuclear data recovered reciprocally monophyletic lineages corresponding to these putative species, and even individuals from the type locality of *P. psorosa* did not form a monophyletic group.

Deviations from species-level monophyly are relatively common in recently diverged taxa and are frequently reported in mtDNA datasets, often because of incomplete lineage sorting or ongoing gene flow (Funk and Omland 2003; Ermakov et al. 2015). Such patterns may be particularly common in phylogenetically young lineages, including Limnephilidae, a family regarded as one of the more recently diversified groups of caddisflies (Schmid 1955; Thomas et al. 2020). However, in the present study, the absence of monophyly was observed not only in mtDNA markers but also in the nuclear marker (wg), suggesting that the lack of genetic structuring cannot be explained solely by stochastic processes affecting mtDNA loci. Furthermore, Haploweb analysis of wg identified a single FFR, indicating that all analysed individuals belong to the same recombining population and that no evidence of reproductive isolation was detected (Flot et al. 2010).

Taken together, the concordant patterns observed across all markers do not support the presence of independently evolving evolutionary lineages within the analysed material. Under the general concept of species lineages, independently evolving lineages are expected to exhibit increasing genetic differentiation and, over time, reciprocal monophyly across independent genetic markers (De Queiroz 2007). However, the incongruence between the originally described morphological differentiation and the molecular patterns observed here is not entirely unexpected, given the complicated taxonomic history of the group (Kolenati 1860; Stein 1874; Mey and Botosaneanu 1985; Oláh et al. 2015). Mey and Botosaneanu (1985) already noted that diagnostic characters partly overlap among neighbouring populations and change gradually across the distribution range. This pattern suggests that the morphological differentiation within the complex may reflect geographic variation rather than discrete taxonomic entities.

Low levels of sequence divergence across all three analysed markers further indicate a shallow evolutionary history of the studied populations. Median uncorrected *p*-distances did not exceed 0.3% for any marker, and maximum divergence remained below 2% even in the two more variable mtDNA markers. Such values fall within the range typically observed for mtDNA intraspecific variation, whereas divergence among species is generally considerably higher and often exceeds ~ 2–3% (Hebert et al. 2003; Kartavtsev 2011; Ratnasingham and Hebert 2013). Similar patterns have been reported for several caddisfly taxa, in which low mtDNA divergence characterises populations of the same species, whereas substantially higher values often indicate genetically differentiated or cryptic species (Hogg et al. 2009; Morinière et al. 2017). Likewise, low divergence was observed in the nuclear marker, a member of the conserved wnt gene family (Siegfried and Perrimon 1994) that has been widely used in phylogenetic studies of insects (Campbell et al. 2000; Wild and Maddison 2008). Although this marker has recently been shown to contain sufficient variation to differentiate closely related caddisfly taxa (Gerwin et al. 2025), such differentiation was not detected in the present dataset. Consistent with these observations, phylogenetic reconstructions showed limited resolution, with shallow genealogies and low support for most nodes.

Strongly negative neutrality test values obtained across the complete mtDNA dataset provide additional insight into the demographic history of the studied complex. Such values are commonly interpreted as signatures of recent demographic expansion or purifying selection (Tajima 1989; Fu 1997; Schmidt and Pool 2002). Similar patterns have been documented in numerous taxa distributed across European mountain systems, including cold-stenotherm montane caddisfly species, such as *Drusus discolor* (Rambur, 1842) (Pauls et al. 2006) and *Rhyacophila pubescens* Pictet, 1834 (Engelhardt et al. 2011), and are often interpreted as evidence of postglacial demographic expansion (Bozáňová et al. 2020; Sworobowicz et al. 2020; Mamos et al. 2021). Climatic oscillations during the Pleistocene likely caused repeated population fragmentation and shifts in species distributions towards southern refugial areas (Hewitt 2003, 2004; Schmitt 2007). However, some cold-adapted species may have persisted in isolated habitats known as extra-Mediterranean refugia located on mountain peaks, along mountain slopes, and in river valleys (Holderegger and Thiel-Egenter 2009; Schmitt and Varga 2012; Pan et al. 2020). Cold-tolerant aquatic insects may have survived close to glaciated regions where fast-flowing streams provided relatively stable habitats associated with the so-called dinodal biome (Malicky 1983; Pauls et al. 2006). Following the retreat of Pleistocene glaciers during the late Quaternary, populations likely expanded rapidly from refugial areas, resulting in shallow genealogies and star-like haplotype networks, as observed in our mtDNA datasets. These historical processes may therefore have contributed to both the shallow genetic structure observed in this study and the subtle variation in male genital morphology within the complex.

Additional insight into the genetic structure of the analysed material is provided by patterns observed in the public barcode database Barcode of Life Data System (BOLD), where our sequences of the *P. psorosa* group cluster within a single Barcode Index Number (BIN; BOLD:AAL1703; https://doi.org/10.5883/BOLD:AAL1703), which represents an approximation of Linnaean species (Ratnasingham and Hebert 2013). However, this BIN also includes sequences identified as several nominal species, including *Pseudopsilopteryx zimmeri* (McLachlan, 1876), *Psilopteryx curviclavata* Botosaneanu, 1957, and *Psilopteryx schmidi* Kumanski, 1970. Rather than indicating true species-level diversity, such BIN sharing may result from biological processes associated with recent divergence, including incomplete lineage sorting or mitochondrial introgression among closely related taxa (Whitworth et al. 2007; Lukhtanov et al. 2009; Ermakov et al. 2015). However, BIN sharing in BOLD is very frequently linked to inconsistencies in morphological identification, particularly in taxonomically challenging groups (Macko et al. 2024; Vuataz et al. 2024; Šamulková et al. 2025), and this may also be the case for some specimens associated with this BIN. This is further supported by the fact that several records attributed to *Pseudopsilopteryx zimmeri* in the BIN are based on larval material, which, according to Waringer et al. (2013), is not readily separable from *Psilopteryx psorosa*. Moreover, the assignment of this BIN to *Pseudopsilopteryx zimmeri* in global databases such as GBIF (https://www.gbif.org/species/1442041) further highlights the need for cautious interpretation of publicly available barcode data. These inconsistencies may also reflect broader challenges in morphological identification within this group and, more generally, at the genus level, where subtle and partly continuous variation in diagnostic characters can complicate species delimitation.

Overall, our results suggest that the two taxa currently recognised as species by Oláh et al. (2015) or as subspecies by Mey and Botosaneanu (1985) most likely represent geographically structured populations rather than distinct evolutionary lineages. Although morphological differentiation of male genitalia has traditionally played a central role in Trichoptera taxonomy, subtle, partly continuous variation may lead to an overestimation of species diversity when unsupported by independent genetic evidence (Dayrat 2005; Padial et al. 2010). The results of the present multilocus analyses therefore suggest that the species-level revisions proposed by Oláh et al. (2015) should be interpreted cautiously. It is worth noting, however, that the gene fragments analysed in this study may not provide sufficient resolution to detect very recent divergence events (Behura 2006). Given the low variability observed in the single nuclear marker (wg), we considered the dataset insufficiently informative for robust coalescent-based species tree analyses. High-resolution markers, such as microsatellites or genome-wide single nucleotide polymorphisms (SNPs), may provide enhanced resolution for detecting fine-scale population structure and recent demographic processes, thereby offering improved resolution for testing recent lineage diversification (Hohenlohe et al. 2021; Schröder et al. 2022). Broader geographic sampling will also be essential, including material from the type localities of the remaining nominal taxa (*P. bohemosaxonica*, *P. harmas* and *P. javorensis*) within the *P. psorosa* group, to evaluate the evolutionary structure of the entire group across the mountain systems of Central Europe. Finally, resolving the species status of this complex will likely require the application of integrative approaches combining detailed morphological analyses, ecological niche modelling, and population genomic data, which may help to better clarify its evolutionary history and taxonomic relationships.

## Conclusions

The results of this study suggest that the morphological characters previously used to delimit the traditionally recognised taxa within the *Psilopteryx psorosa* species group, whether treated as subspecies by Mey and Botosaneanu (1985) or as species by Oláh et al. (2015), likely reflect historical geographic differentiation among populations rather than distinct evolutionary lineages. The lack of clear genetic structure across both mtDNA and nuclear markers further supports the interpretation that the analysed populations represent a single evolutionary lineage, corresponding to a single Linnaean species. Further resolution of diversification within this group will require broader geographic sampling and the application of higher-resolution genomic markers, ideally combined with detailed morphological and ecological analyses within an integrative taxonomic framework.

## Additional information

### Artificial Intelligence (AI) use

The authors accept full responsibility for the content of the manuscript, including the disclosure of any use of AI.

No AI tools were used in the preparation of this manuscript.

### Author ORCIDs

M. Šamulková https://orcid.org/0009-0000-9599-5255

T. Navara https://orcid.org/0009-0006-8314-0480

P. Chvojka https://orcid.org/0000-0003-0946-0540

Z. Čiamporová-Zaťovičová https://orcid.org/0000-0003-0506-6212

P. Macko https://orcid.org/0009-0008-0714-7490

### Data availability

All DNA sequences generated in this study are publicly available in the Barcode of Life Data System (BOLD) under the dataset DS-TRIPSILO (https://doi.org/10.5883/DS-TRIPSILO) and in GenBank under accession numbers PZ471246–PZ471303 (COI), PZ471188–PZ471245 (cytB), PZ471130–PZ471187 (Wnt1).

## Supplementary materials

Supplementary material 1 Occurences Data type xlsx Explanation note List of sampling sites and their characteristics processed in this study. https://binary.pensoft.net/file/1704856This dataset is made available under the Open Database License (http://opendatacommons.org/licenses/odbl/1.0/). The Open Database License (ODbL) is a license agreement intended to allow users to freely share, modify, and use this Dataset while maintaining this same freedom for others, provided that the original source and author(s) are credited. Michaela Šamulková, Tomáš Navara, Pavel Chvojka, Zuzana Čiamporová-Zaťovičová, Patrik Macko

Supplementary material 2 PCR conditions Data type xlsx Explanation note PCR reaction composition, thermal cycling conditions, and primer sequences for COI, cytB, and wg markers. https://binary.pensoft.net/file/1704857This dataset is made available under the Open Database License (http://opendatacommons.org/licenses/odbl/1.0/). The Open Database License (ODbL) is a license agreement intended to allow users to freely share, modify, and use this Dataset while maintaining this same freedom for others, provided that the original source and author(s) are credited. Michaela Šamulková, Tomáš Navara, Pavel Chvojka, Zuzana Čiamporová-Zaťovičová, Patrik Macko

Supplementary material 3 Maximum likelihood phylogenetic trees Data type tiff Explanation note Maximum likelihood phylogenetic trees based on two mitochondrial (COI, cytB) markers and one nuclear marker (wingless). https://binary.pensoft.net/file/1704858This dataset is made available under the Open Database License (http://opendatacommons.org/licenses/odbl/1.0/). The Open Database License (ODbL) is a license agreement intended to allow users to freely share, modify, and use this Dataset while maintaining this same freedom for others, provided that the original source and author(s) are credited. Michaela Šamulková, Tomáš Navara, Pavel Chvojka, Zuzana Čiamporová-Zaťovičová, Patrik Macko

Supplementary material 4 Bayesian inference phylogenetic trees Data type tiff Explanation note Bayesian inference phylogenetic trees based on two mitochondrial markers (COI, cytB) and one nuclear marker (wingless). https://binary.pensoft.net/file/1704859This dataset is made available under the Open Database License (http://opendatacommons.org/licenses/odbl/1.0/). The Open Database License (ODbL) is a license agreement intended to allow users to freely share, modify, and use this Dataset while maintaining this same freedom for others, provided that the original source and author(s) are credited. Michaela Šamulková, Tomáš Navara, Pavel Chvojka, Zuzana Čiamporová-Zaťovičová, Patrik Macko
