# Supplementary figures and images for: Multilocus re-evaluation of species boundaries in the Central European Psilopteryx psorosa species group (Trichoptera, Limnephilidae) with shallow morphological differentiation
Source: Zookeys. 2026 Jul 1;1283:323–42. doi: 10.3897/zookeys.1283.193317 (PMC13347112; doi:10.3897/zookeys.1283.193317)

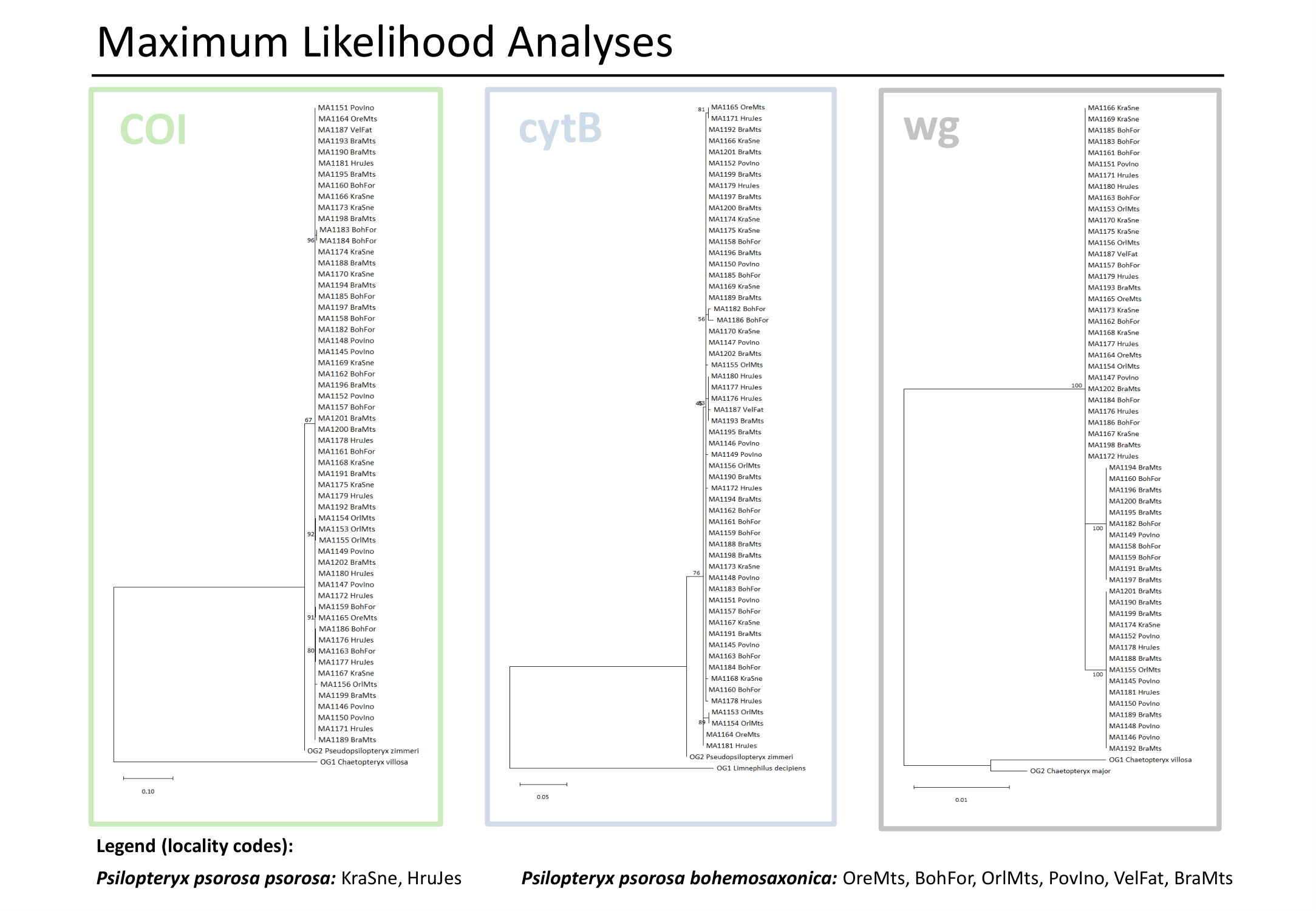

Supplement: Supplementary material 3 — Maximum likelihood phylogenetic trees [file zookeys-1283-323_article-193317__-s003.tiff]

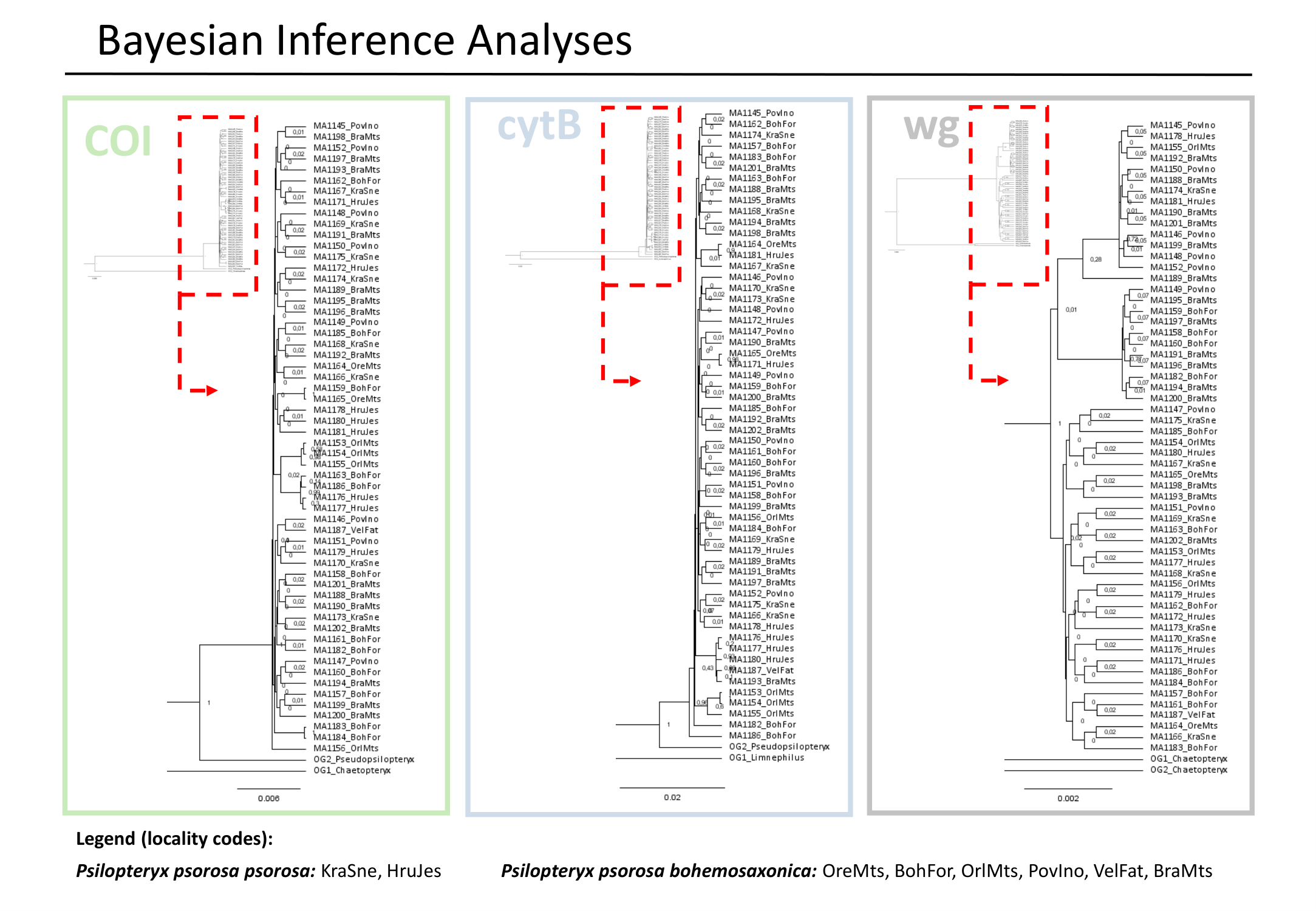

Supplement: Supplementary material 4 — Bayesian inference phylogenetic trees [file zookeys-1283-323_article-193317__-s004.tiff]
